# Progesterone Signaling and Mammalian Ovarian Follicle Growth Mediated by Progesterone Receptor Membrane Component Family Members

**DOI:** 10.3390/cells11101632

**Published:** 2022-05-13

**Authors:** John J. Peluso

**Affiliations:** 1 Department of Cell Biology, University of Connecticut Health Center, Farmington, CT 06030, USA; peluso@uchc.edu; 2 Department of Obstetrics and Gynecology, University of Connecticut Health Center, Farmington, CT 06030, USA

**Keywords:** apoptosis, follicle development, mitosis, progesterone, progesterone receptor membrane component

## Abstract

How progesterone influences ovarian follicle growth is a difficult question to answer because ovarian cells synthesize progesterone and express not only the classic nuclear progesterone receptor but also members of the progestin and adipoQ receptor family and the progesterone receptor membrane component (PGRMC) family. Which type of progestin receptor is expressed depends on the ovarian cell type as well as the stage of the estrous/menstrual cycle. Given the complex nature of the mammalian ovary, this review will focus on progesterone signaling that is transduced by PGRMC1 and PGRMC2 specifically as it relates to ovarian follicle growth. PGRMC1 was identified as a progesterone binding protein cloned from porcine liver in 1996 and detected in the mammalian ovary in 2005. Subsequent studies focused on PGRMC family members as regulators of granulosa cell proliferation and survival, two physiological processes required for follicle development. This review will present evidence that demonstrates a causal relationship between PGRMC family members and the promotion of ovarian follicle growth. The mechanisms through which PGRMC-dependent signaling regulates granulosa cell proliferation and viability will also be discussed in order to provide a more complete understanding of our current concept of how progesterone regulates ovarian follicle growth.

## 1. Introduction

It has been over 40 years since Dr. Irving Rothchild advanced the concept that progesterone regulates its own secretion from the corpus luteum. In words taken from Dr. Rothchild’s extensive review of 1981 [1], “the possibility that progesterone may have a stimulatory effect on its own secretion is based on a rather assorted collection of [descriptive] findings”. Fifteen year later, Rothchild [2] concludes that “the system through which progesterone controls its own secretion is still undiscovered”. In fact, studies with the progesterone receptor antagonist RU486 revealed that it could either raise or lower progesterone secretion depending on the experimental conditions, thereby adding to the confusion.

Similarly, descriptive studies conducted in the 1970s and 1980s on hypophysectomized gonadotropin treated hamsters [3] and gonadotropin-treated hamsters [4], rats [5], mice [6], and monkeys [7] demonstrated that progesterone inhibits follicle growth. In vitro studies also revealed that progesterone acts directly on granulosa cells of mice [6], rats [8,9,10], and women [11,12] to slow the rate of mitosis and to preserve granulosa cell viability. These findings are consistent with the detection by Schreiber’s group of an unknown progesterone binding protein that was present in the ovaries of estrogen-stimulated hypophysectomized immature female rats [13,14].

While studies on the effects of progesterone on the mitosis and survival of granulosa cells were being conducted, various research groups were simultaneously characterizing a progesterone binding protein isolated from chicken oviducts [15,16,17,18]. These studies ultimately lead to the characterization and cloning of the nuclear progesterone receptor (PGR) [19,20]. The cloning of the PGR in the mid 1980s allowed for the development of probes to detect PGR by Northern blot and later by qPCR as well as the generation of antibodies to detect both the A and B forms of PGR. Studies using these reagents revealed that in mice and rats PGR was transiently expressed for just a few hours in granulosa cells of preovulatory follicles during the ovulatory luteinizing hormone surge [21,22,23,24,25]. Prior to the publications on PGR expression, it was assumed that the progesterone binding protein detected by Schreiber’s group in 1979 was PGR. However, the temporal pattern of PGR expression made it clear that the progesterone binding proteins detected by Schreiber’s group were not PGR and thus raised the question as to how progesterone could regulate granulosa cell mitosis and survival if granulosa cells of developing follicles do not express PGR.

These types of paradoxical and sometimes contradictory findings related to progesterone’s actions in the corpora lutea and granulosa cells reinforced Rothchild’s conclusion that these “paradoxes often if not always tell us that there is something we do not know” [2]. That something could be that there are other receptors that mediate progesterone’s action but investigating the nature of putative alternative progesterone receptors was pushed to the back burner as work on PGR’s mode of action dominated the research effort in this area for decades.

Although elucidating PGR’s mechanism of action was the major research focus, limited studies were also being conducted to identify alternative receptors for progesterone. Evidentially, these studies identified two other families of receptors that could potentially mediate progesterone’s action within the ovary: the progestin and adipoQ (PAQR) receptor family and the progesterone membrane receptor component family (PGRMCs). Members of each of these families of receptors have been detected in mammalian ovaries. Because PGR’s mode of action in regulating ovarian function will be discussed by Dr. Rebecca Robker and PAQR signaling and reproductive function will be addressed by Dr. Peter Thomas, this review will focus on the roles that PGRMC1 and PGRMC2 play in regulating mammalian ovarian follicle development.

## 2. Search for an Alternative Progesterone Receptor in Granulosa Cells

Detecting specific progesterone binding in ovarian extracts demonstrated the presence of a progesterone binding protein in 1979 [13,14]. Similar ligand binding studies were also conducted using spontaneously immortalized granulosa cells (SIGCs) that were developed by Dr. Robert Burghardt and derived from granulosa cells of rat preovulatory follicles [26]. These cells were capable of specifically binding ^3^H-progesterone which cannot be displaced by dexamethasone, or RU486 [27]. Since RU486 binds to PGR at a higher affinity than P4 [28], the inability of RU486 to reduce progesterone binding to SIGCs [27] and to attenuate progesterone’s action provided further support for the existence of other receptors for progesterone in SIGCs.

These observations also provided a rationale for using an antibody directed against the progesterone binding site of PGR to search for a putative receptor that could mediate progesterone’s action. Western blot analysis using this antibody, referred to as C-262, detected a 50–60 kDa protein in lysates of rat ovaries as well as rat granulosa cells and SIGCs [29]. Subsequent studies suggested that this C-262-detected protein was involved in mediating progesterone’s actions [30] so an attempt to identify it was made using C-262-based affinity column chromatography [31]. This approach detected a protein initially referred to as hypothetical protein CGI-55, subsequently referred to as RDA-288 and ultimately known as either Plasminogen Activator Inhibitor 1 RNA-binding protein (PAIRBP1) or Serpine mRNA Binding Protein 1 (SERBP1) [31]. An analysis of the sequence of PAIRBP1 failed to provide any insight into how it could be involved in mediating progesterone’s action as it did not even have a transmembrane domain or kinase binding sites. Again, the words of Irving Rothchild rang true “there is something we do not know” [2]. To try to identify that something, an antibody was generated to PAIRBP1 and used to isolate proteins that interact with PAIRBP1. This approach identified progesterone receptor membrane component 1 (PGRMC1) as a protein that interacted with PAIRBP1 in SIGCs [32]. Several years earlier, PGRMC1 had been isolated from porcine liver membranes and cloned by Dr. Martin Wehling’s group [33,34]. Moreover, PGRMC1 was shown to be present in membrane fractions as a ≈28 and ≈56 kDa protein that bound progesterone through high and low affinity binding sites with K_d_ of 11 and 286 nM, respectively [34]. These characteristics suggested that if PGRMC1 and possibly PGRMC2, the second PGRMC family member to be cloned [35], were expressed in ovarian cells, they would be good candidates to be mediators of progesterone’s anti-mitotic and anti-apoptotic actions in granulosa cells.

### 2.1. Ovarian Expression of PAIRBP1 and PGRMC Family Members

#### 2.1.1. Expression in Ovaries of Laboratory and Domestic Animals

**Figure 1:**
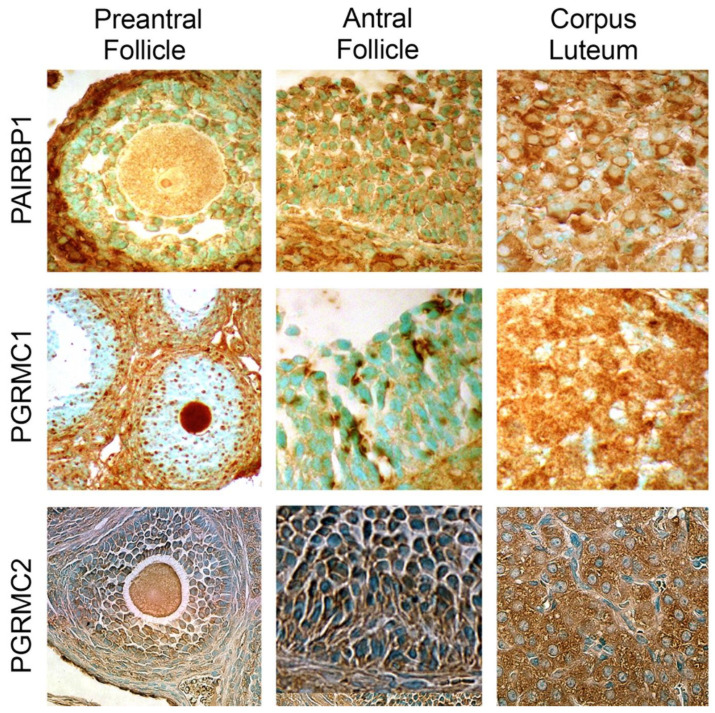
Localization of PAIRBP1, PGRMC1, and PGRMC2 in preantral and antral follicles as well as cells of the corpus luteum. Each protein is detected as a reddish-brown stain. Images for PAIRBP1 and PGRMC1 illustrate their localization in rat ovaries and were taken from Peluso et al. [36], while the images of PGRMC2 reveal its localization in mouse ovaries and are taken from Griffin et al. [39].

The first study on the expression of PAIRBP1 and PGRMC1 (Figure 1) was conducted on immature rat ovaries. This study showed that these two proteins were expressed in granulosa cells of preantral and antral follicles as well as the associated thecal cells and surface epithelial cells [36]. PAIRBP1 in both preantral and antral follicles was always associated with the plasma membrane and cytoplasm of the granulosa cells, while PGRMC1 was most predominately detected in the nucleus of granulosa cells with light staining in the cytoplasm/plasma membrane of growing preantral follicles. This distribution changed to more membrane localization in the larger antral follicles [36] whose granulosa cells were less frequently undergoing mitosis [37]. In addition, PGRMC1 and PAIRBP1 expression in luteal cells was greater than that observed in granulosa cells as judged by both immunohistochemical and Western blot analysis [36]. Similar expression profiles were observed in mouse ovaries [38]. PGRMC1 was also present in the cumulus cells and oocytes [36]. PGRMC2 showed a similar pattern of expression in oocytes, granulosa, thecal, and surface epithelial cells with the most pronounced staining observed in corpora lutea (Figure 1) [39,40]. Similar to PAIRBP1, PGRMC2 was not detected in the nuclei of any ovarian cell type [39].

Although not as extensively examined as in the rat ovary, PGRMC1 was detected in the porcine and bovine cumulus cells [41,42], granulosa cells [43,44], and luteal cells [45,46,47,48]. PGRMC1 was also detected in the luteal cells of domestic cat and lynx corpora lutea [49]. Taken together, these studies imply that PGRMC family members could be involved in regulating ovarian function of numerous species.

#### 2.1.2. Expression in the Healthy and Dysfunctional Human Ovary

There is only one study that showed that PGRMC1 is expressed in non-human primate corpora lutea [50]. However, there are several studies in which PGRMC1 was detected in human ovarian cells. The first study was by Engmann et al. [51] in which the majority of granulosa/luteal cells harvested from the ovaries of women who had undergone gonadotropin-induced ovarian stimulation as part of their in vitro fertilization (IVF) infertility treatment were shown to express PGRMC1 as well as its binding partner PAIRBP1. Interestingly, gonadotropin-induced follicle development was reduced in patients with elevated granulosa/luteal cell levels of PGRMC1 mRNA [52], suggesting that PGRMC1 plays an ill-defined role in regulating follicle growth in women. Additional support for a role of PGRMC1 in regulating follicle development in women came from the finding that women experiencing premature ovarian insufficiency had lower levels of PGRMC1 in their peripheral leukocytes [53] and/or a functional mutation in PGRMC1 [53,54]. Lower levels of PGRMC1 were also detected in the peripheral leukocytes of women with polycystic ovary syndrome (PCOS), suggesting that decreased PGRMC1 levels in women with PCOS is an indication of altered follicle development and reduced ovulatory capacity [55]. Similarly, PGRMC1 and PGRMC2 levels in peripheral leukocytes were shown to be decreased after controlled gonadotropin stimulation protocol. However, PGRMC1 levels were not related to the number of follicles that were induced to develop [56], so the significance of the changes in PGRMC1 levels observed in peripheral leukocytes as it relates to ovarian function is not clear.

While these expression studies are important, they simply demonstrate that PGRMC1 and in some cases PGRMC2 and PAIRBP1 are expressed in various ovarian cell types of all species studied. They do not, however, demonstrate a causal relationship between progesterone’s ability to regulate ovarian function through a pathway mediated through PGRMC1 and/or PGRMC2.

## 3. PGRMC1 as a Mediator of Progesterone’s Actions

To demonstrate that PGRMC family members are involved in regulating ovarian function, studies must show that PGRMC1 specifically binds progesterone and that PGRMC1 and PGRMC2 are required for progesterone’s ability to regulate ovarian cell function. The following sections provide evidence that PGRMC1 and PGRMC2 are essential components of the signaling mechanism that mediates progesterone’s actions in ovarian cells.

### 3.1. Capacity of PGRMC1 to Specifically Bind Progesterone

Three approaches were used to demonstrate that PGRMC1 specifically binds progesterone. The first approach used SIGCs that do not express PGR. These studies revealed that SIGCs specifically bound ^3^H-progesterone and that overexpressing PGRMC1 increased specific ^3^H-progesterone [36]. In addition, depleting PGRMC1 greatly reduced the ability of SIGCs to bind ^3^H-progesterone [57] but depleting either PAIRBP1 [58] or PGRMC2 [39] did not infer with ^3^H-progesterone binding.

**Figure 2:**
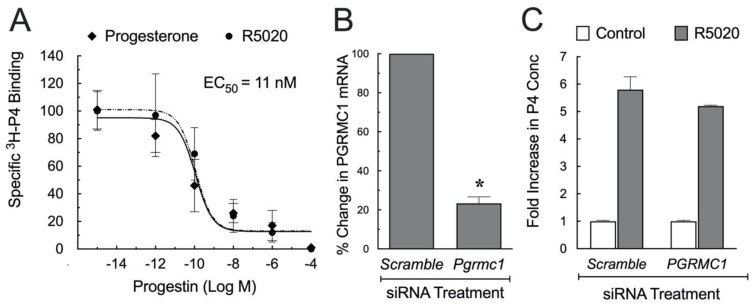
P4 binding characteristic of partially purified human PGRMC1. hGL5 cells were transfected with either an empty vector that encoded GFP or a vector that encoded a GFP-human PGRMC1 fusion protein. Ligand binding studies revealed that both P4 and R5020 displaced ^3^H-progesterone with the same dose-dependent characteristics. Curve fitting the binding data revealed an EC_50_ of 11 nM for both P4 and R5020 (**A**). Panel (**B**) shows the effect of siRNA treatment on the levels of PGRMC1 mRNA; * indicates a significant decrease from scramble siRNA (*p* < 0.05). The effect of R5020 on P4 secretion after 3 days of treatment with either scramble or PGRMC1 siRNA is shown in Panel (**C**). Data taken from Peluso et al. [62].

The second approach used SIGCs that were transfected with an expression vector that encoded a GFP-PGRMC1 fusion protein. The GFP-PGRMC1 fusion protein was then isolated and assessed for its capacity to bind ^3^H-progesterone. This study showed that GFP-PGRMC1 specifically binds progesterone with K_d_ of ≈ 35 nM and studies in which various deletion mutants of GFP- porcine PGRMC1 fusion protein indicated that each domain of PGRMC1 was required for maximum ^3^H-progesterone binding. Further insight into the capacity of human PGRMC1 to bind progesterone was provided by studies that assessed the capacity of a missense point mutation (H165R) of PGRMC1 which was observed in women with premature ovarian insufficiency. This mutation although having reduced ability to preserve cell viability, bound ^3^H-progesterone with the same affinity (EC_50_ ≈ 11 nM) as the wild-type PGRMC1 (Figure 2A) [53]. These findings differ from the initial studies conducted using purified porcine liver membranes that detected both a high affinity and a low affinity progesterone binding site but this study did not specifically demonstrate that both bindings sites were due to the presence of PGRMC1 in this preparation [34]. The third approach was to express and purify PGRMC1 from *E. coli* and monitor progesterone binding activity using UV-vis and resonance Raman spectroscopies. This approach provided direct evidence of PGRMC1′s capacity to specifically bind progesterone [59].

While the previously described studies conclusively demonstrate that PGRMC1 binds ^3^H-progesterone, an interaction may exist with another progestin binding protein, PAQR7, that could enhance ^3^H-progesterone binding. This possibility is supported by the finding that overexpression of PGRMC1 increases the amount of both PGRMC1 and PAQR7 that localizes to the plasma membranes [60]. Unfortunately, this study did not reveal the amount of progesterone that bound to PGRMC1 verses PAQR7. PGRMC1 does interact with PAQR7 [61] and depleting PAQR7 limits the effectiveness of progesterone’s action [61] but the mechanism by which depleting PAQR7 influences the biological actions of progesterone in ovarian cells still remains to be determined.

### 3.2. PGRMC Family Members and Their Roles in Regulating Granulosa Cell Function

In order for ovulatory follicles to develop, granulosa cells must regulate three major functions: steroidogenesis, proliferation, and maintenance of cell viability. Given its expression profile and capacity to interact with so many different proteins, PGRMC1 and PGRMC2 could influence each of these functions as outlined in the following sections.

#### 3.2.1. Steroidogenesis

As indicated in the introduction, progesterone can stimulate its own secretion but how it mediates its action is not known. Both PGRMC1 and PGRMC2 interact with various cytochrome P450 enzymes. Some of these enzymes stimulate steroid synthesis [63] primarily by activating Cytochrome P450 51A1, thereby promoting cholesterol biosynthesis which is required for progesterone synthesis as well as the synthesis of estrogen and testosterone. Further, depleting PGRMC1 in HEK293 cells blocks cholesterol synthesis [64], making it possible that PGRMC1 mediates progesterone’s ability to stimulate progesterone biosynthesis by facilitating cholesterol synthesis. To test this possibility, a series of studies were conducted using hGL5 cells, a line derived from human granulosa/luteal cells obtained from women undergoing IVF. These cells express PGR, PAQR-7, -8, and -5, as well as PGRMC1 [62]. For these studies, the synthetic progestin, R5020, was used to stimulate progesterone secretion as it is not detected in the ELISA assay of progesterone. Not only does R5020 bind to PGR [65] and PAQRs [66], it also binds human PGRMC1 with relatively high affinity similar to that of progesterone (Figure 2A) [62]. To assess its role in regulating progesterone synthesis, PGRMC1 was depleted from hGL5 using siRNA (Figure 2B) and the cells treated with R5020. Scramble siRNA was used a control and R5020 induced a 5-fold increase in progesterone secretion within 3 h of culture and depleting PGRMC1 did not alter R5020′s capacity to stimulate the secretion of progesterone (Figure 2C) [62]. It appears then that PGRMC1 is not required for progesterone to promote its own secretion. This finding is consistent with the observation that ablating PGRMC1 globally only lowered serum cholesterol levels by 8% [67]. Since PGRMC1 is also ablated from the ovaries of global PGRMC1 knockout mice [68] and cholesterol that is required for ovarian progesterone synthesis is derived from both serum and de novo synthesis [69], it is likely that depleting PGRMC1 did not deplete cholesterol sufficiently to interfere with progesterone synthesis. To further test this concept, progesterone levels were measured in the serum of conditional knock out mice designed to ablate either PGRMC1 [38] or PGRMC2 [40] from the corpora lutea, the major site of progesterone synthesis. This study revealed that serum progesterone levels of the conditional knockout mice were the same as control. Thus, it is unlikely that PGRMC1 or PGRMC2 has influenced progesterone synthesis and so it remains as in Rothchild’s words that “there is something about how progesterone stimulates its synthesis that we still do not know” [2].

#### 3.2.2. Mitosis and Apoptosis

As previously discussed, progesterone inhibits ovarian follicle growth by slowing the rate at which granulosa cells undergo mitosis. Defining the mechanisms that regulate granulosa cells mitosis is complicated by the fact that granulosa cells tend to differentiate in vitro and as a result they lose their capacity to proliferate. In addition, developing follicles have two populations of granulosa cells: small and large granulosa cells, which can be isolated by percoll gradient centrifugation [70]. Only the small granulosa cells proliferate efficiently in vitro [70]. However, short-term culture studies of small granulosa cells revealed that various growth factors including insulin stimulate rat granulosa cell mitosis and progesterone inhibits insulin-induced granulosa cell mitosis [13]. This anti-mitotic action of progesterone was not mimicked by testosterone, dihydrotestosterone, or dexamethasone [9]. Finally, the PGR antagonist, RU486, did not enhance insulin-induced mitosis [9]. The inability of RU486 to potentiate insulin’s mitogenic effect fits well with the findings that these cells do not express PGR [21,22,23,24,25]. Studies using cultured human granulosa cells confirmed that progesterone suppresses proliferation [11,12].

**Figure 3:**
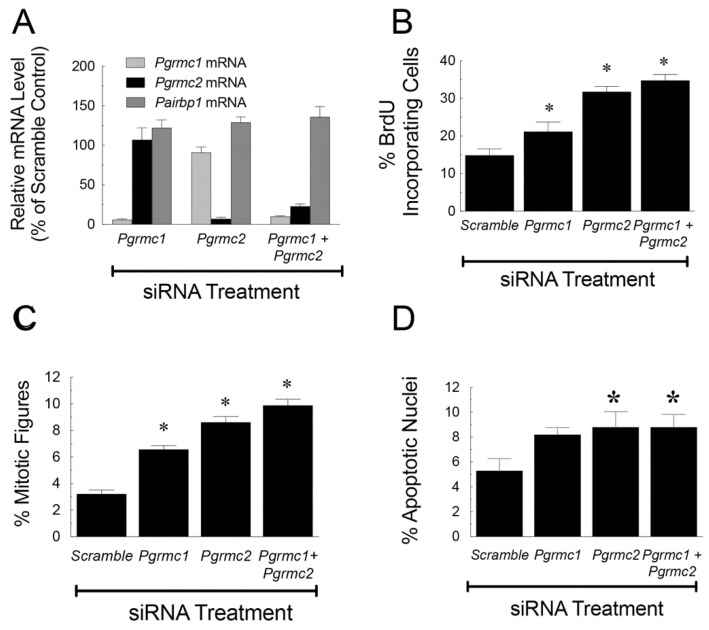
The effect of *Pgrmc1* and/or *Pgrmc2* siRNA treatment on the mRNA levels of *Pgrmc1*, *Pgrmc2*, and *Pairbp1* (**A**); percentage of cells incorporating BrdU (**B**); percentage of cells arresting in metaphase (**C**); and the percentage of apoptotic nuclei (**D**). The values are means ± standard error. * Value in this and other figures is significantly different from scramble control (*p* < 0.05). Data taken from Peluso et al. [71].

While the data from short-term cultures of small rat granulosa cells support the concept that progesterone inhibits granulosa cell mitosis, having to use percoll gradient centrifugation to isolate a limited number of small granulosa cells made it difficult to elucidate the molecular pathways through which progesterone mediates its anti-mitotic action. In order to determine whether PGRMC1 is required for progesterone to inhibit proliferation, studies were conducted using SIGCs. Progesterone suppressed SIGC proliferation and like granulosa cells, SIGCs expressed both PGRMC1 and PGRMC2 but not PGR [71]. Furthermore, treatment with siRNAs selectively depleted PGRMC1 and PGRMC2 without altering PAIRBP1, a known binding partner of PGRMC1 (Figure 3A). Depleting PGRMC1 and/or PGRMC2 resulted in an increase in the percentage of cells entering the cell cycle as judged by the percentage of cells incorporating bromodeoxyuridine (BrdU) (Figure 3B) [71]. Cells depleted of PGRMC1 and/or PGRMC2 either arrested in metaphase (Figure 3C) or underwent apoptosis (Figure 3D) [71]. The failure to complete mitosis and undergo apoptosis was dramatically illustrated by time-lapse photography of bovine granulosa cells in which PGRMC1 was depleted [72]. Taken together, these studies suggest that PGRMC1 and PGRMC2 prevent inappropriate entry into the cell cycle and thereby prevent granulosa cells from undergoing apoptosis.

While depleting PGRMC1 and/or PGRMC2 leads to premature or inappropriate entry into the cell cycle, overexpression of either of these two PGRMC family members suppresses entry into the cell cycle. This finding although consistent with the proposed function of PGRMC1 and PGRMC2 raises the question as to how the anti-mitotic action of PGRMC1 and PGRMC2 can be overcome in order to allow for cell proliferation. The answer to this question appears to be that the expression of PGRMC2 is transiently decreased prior to entry into the cell cycle and then increased again in the G_2_/M stage of the cell cycle [39]. The factors that regulate the expression of PGRMC2 in a cell cycle-dependent manner remain to be determined and should be a focus of future studies with the mostly likely candidates being ovarian growth factors.

As previously discussed, data derived from SIGCs and bovine granulosa cells clearly illustrate a role for PGRMC1 and PGRMC2 in progesterone’s ability to regulate the entry into the cell cycle while maintaining cell viability. However, SIGC-based studies are limited in that SIGCs do not express PGR; thus cannot reveal a complete picture of progesterone’s role in regulating human granulosa cells which do express PGR and PAQR7. To address this issue, human granulosa/luteal cells were isolated from 10 patients undergoing IVF and maintained in culture. Human granulosa/luteal cells from all of these patients expressed PGRMC1, PGRMC2, PAQR7, and PGR with PGRMC1 mRNA being most abundant, followed by PAQR7, PGRMC2, and PGR [61]. These cells also express PAQR5 and PAQR8 [62]. However, granulosa/luteal cells from each patient had a slightly different expression profile of these progesterone receptors. Whether the expression profile determines the overall response to progesterone is unknown, adding an additional layer of complexity in defining progesterone’s action in human granulosa cells.

**Figure 4:**
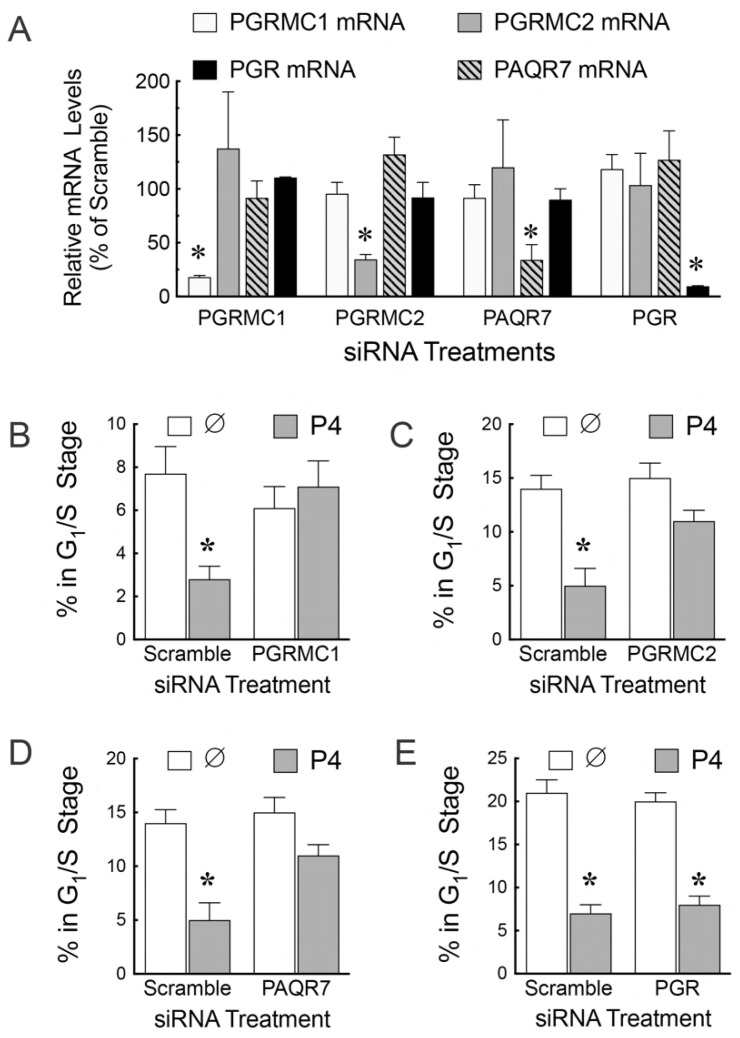
The effect of *Pgrmc1*, *Pgrmc2,* siRNA treatment on the mRNA levels of *Pgrmc1*, *Pgrmc2*, *Paqr7*, and Pgr. (**A**). The effect of progesterone (P4) and PGRMC1 siRNA (**B**), PGRMC1 siRNA (**C**), PAQR7 siRNA (**D**), and PGR siRNA (**E**) on the percentage of cells entering the G_1_/S stage of the cell cycle as assessed using the Fucci cell cycle indicator. Data taken from Sueldo et al. [61]. The ∅ identifies the no treatment control group. * indicates that P4 induced a significant decrease compared to the no treatment control.

To investigate the capacity of each of these proteins to mediate progesterone’s ability to suppress entry into the cell cycle, each was depleted using specific siRNA (Figure 4A). Progesterone’s ability to suppress entry into the cell cycle was assessed using the FUCCI cell cycle indicator. This study demonstrated that progesterone’s ability to suppress entry into the cell cycle was dependent on PGRMC1 (Figure 4B), PGRMC2 (Figure 4C), and PAQR7 (Figure 4D) but not PGR (Figure 4E) [61]. Interestingly PGRMC1, PGRMC2, and PAQR7 interact with each other apparently forming a complex within the cytoplasm that is required for progesterone to limit entry into the cell cycle [61]. The exact role that each of these proteins plays in mediating progesterone’s ability to restrict entry into the cell cycle remains to be determined.

#### 3.2.3. Duration of Metaphase

Progesterone not only suppresses entry into the cell cycle [61,71] but it also increases the percentage of SIGCs detected in the G_2_/M stage of the cell cycle, which accounts in part for a decrease in the number of cells present after 48 h of culture [72]. This suppression of cell cycle traverse is reflected in the increase in the percentage of mitotic figures present after being cultured in progesterone-supplemented media [72]. The prolongation of mitosis seems to be dependent on progesterone’s interaction with PGRMC1 which increases the stability of the mitotic spindle and in turn slows the progression through the metaphase stage of the cell cycle [73]. This is likely due to the PGRMC1–ß-tubulin interaction that develops within the mitotic spindle. Whether progesterone’s ability to slow the progression through metaphase is advantageous or deleterious has not been assessed and may depend on the duration and level of progesterone exposure and how this affects the interaction between PGRMC1 and Aurora kinase B [72].

Taken together, these observations support the concept that progesterone acting through PGRMC1 and PGRMC2 restricts cell cycle traverse to times when conditions are optimal for the successful completion of mitosis. This is important in that it avoids a mitotic catastrophe that results in apoptosis. While this is a central aspect of progesterone-PGRMC signaling, it may not be the only mechanism through which progesterone prevents apoptosis. Progesterone-PGRMC1 signaling also functions to limit oxidative-stress-induced apoptosis. This is illustrated by studies using human granulosa/luteal cells in which progesterone inhibits apoptosis induced by exposure to the oxidative stressor, hydrogen peroxide. These studies showed that depleting either PGRMC1 or PGRMC2 limits progesterone’s ability to inhibit hydrogen peroxide-induced apoptosis [74]. AG 205, a PGRMC1 antagonist, also blocked progesterone’s ability to prevent hydrogen peroxide-induced apoptosis but did not reduce the levels of PGRMC1. Rather, AG 205 increased the proportion of monomeric forms of PGRMC1 and reduced the proportion of high molecular weight oligomers [75]. The higher molecular weight forms of PGRMC1 predominately localize to the nucleus [75,76,77] and increase the expression of genes known to inhibit apoptosis [75,76,77] will be discussed in the following section.

## 4. PGRMC1 and Multiple Sites of Action

It is clear that PGRMC family members play essential roles in regulating progesterone’s effects on granulosa cell mitosis and viability and in turn follicle growth. What is equally clear is that we only have hints as to how PGRMC1 and PGRMC2 mediate their action. We know most about the mechanism of action of PGRMC1. For example, we know that PGRMC1 localizes to three cellular sites: (1) the plasma membrane, (2) the cytoplasm including but not limited to membranes of endoplasmic reticulum, and (3) the nucleus. PGRMC1 also exists as a monomer (≈25 kDa) and oligomers (≥50 kDa) with oligomers formed in part due to heme-dependent interaction [78]. The monomers localize to the membrane fraction [77]. Some of the oligomers are sumoylated with the level of sumoylation increased by progesterone [77]. Although sumoylated PGRMC1 localizes to the nucleus, sumoylation is not required for entry into the nuclei as PGRMC1 with mutated sumoylation sites are still detected within the nucleus [77]. PGRMC1 can also be phosphorylated. These characteristics allow for the possibility that PGRMC1 has distinct modes of action depending on its cellular localization and its sumoylation and phosphorylation status, but considerably more studies on how posttranslational modifications influence PGRMC1′s cellular localization and function are needed. The following sections outline putative mechanisms that originate at each point of PGRMC1′s cellular localization.

### 4.1. Actions Initiated at the Plasma Membrane

As suggested by its name, some molecules of PGRMC1 localize to the plasma membrane. This was confirmed by both confocal imaging and the detection of PGRMC1 among the proteins isolated from the plasma membranes of ovarian cells [36]. It was also known that progesterone signaling in ovarian cells activates a protein kinase G pathway that is likely mediated in part via PGRMC1 [79,80,81]. Other kinase pathways such as those mediated by phosphoinositide 3-kinase (PI3K), protein kinase B (PKB; as known as AKT) and mitogen-activated protein kinase (MAPK) have also been associated with PGRMC1′s mode of action in non-ovarian tissue. However, evidence for coupling PGRMC1 to G-proteins that activate these kinases has been difficult to obtain. One possible mechanism to explain the PGRMC1-kinase relationship is that PGRMC1 increases the levels of PAQR7 [60], the insulin receptor [82] and the epidermal growth factor (EGF) receptor [83] that are present at the plasma membrane. The delivery of the EGF receptor is dependent on the dimeric form of PGRMC1 [83]. Although ovarian cells express all three of these receptors, whether PGRMC1 directs them to the plasma membranes of ovarian cells has not been determined and merits further investigation. Regardless, PGRMC1′s ability to direct these receptors to the plasma membrane could facilitate the activation of the PI3K/AKT and MAPK pathways which regulate the activity of various cell-cycle-dependent kinases that drive mitosis [84]. Whether progesterone binding to PGRMC1 alters its ability to direct these receptors to the plasma membrane is not known but if progesterone limits the number of growth factors receptors at the plasma membrane this would represent a potential mechanism through which progesterone limits cell proliferation. Finally, it is not clear whether PGRMC1′s sole function is to direct other receptors to the plasma membrane. It is also possible that it directly participates in signal transduction pathways independent of other receptors. This is an important question that should be addressed in the future.

Interestingly, studies indicate that MAPK can activate transcription factors such as ternary complex factors (e.g., Tcf/Lef1) and can ultimately result in the transcription of immediate-early genes that induce mitosis [85]. Progesterone suppresses Tcf/Let1-dependent transcriptional activity through a PGRMC1-dependent manner [77] which could account in part for progesterone’s anti-mitotic activity. How the phosphorylation and sumoylation status of PGRMC1 influences progesterone’s ability to suppress Tcf/Lef1 transcriptional activity and ultimately its ability to suppress mitosis and apoptosis remains to be determined but could involve progesterone’s ability to suppress MAPK activity [86].

### 4.2. Actions Initiated within the Cytoplasm

**Figure 5:**
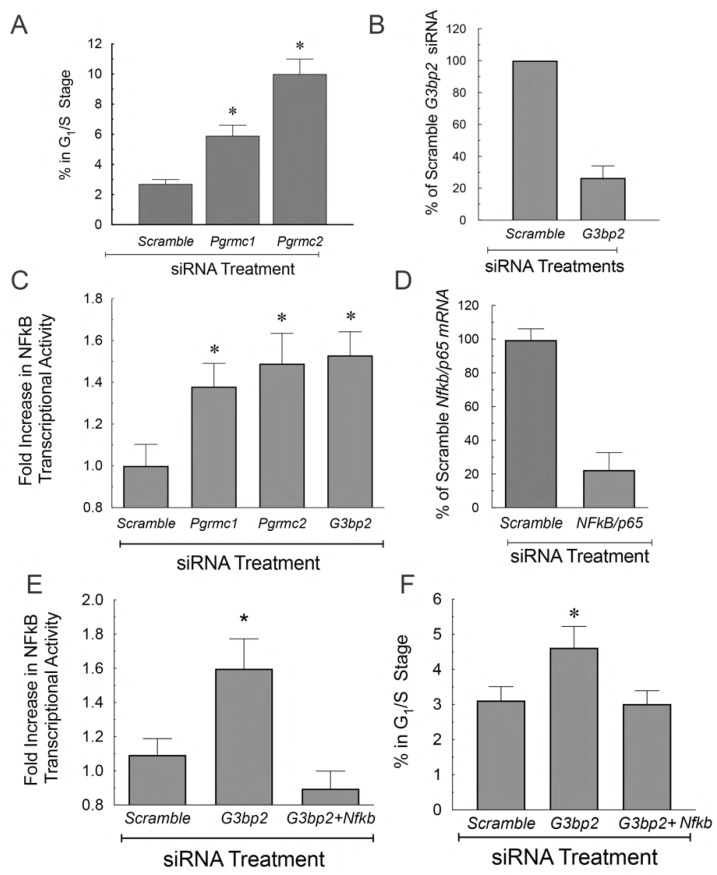
The effect of *Pgrmc1* and *Pgrmc2* siRNA treatment on the percentage of SIGCs entering the cell cycle (**A**). Panel (**B**) shows the effect of G3BP2 siRNA on the mRNA levels of G3BP2. The effect of *Pgrmc1*, *Pgrmc2*, and G3BP2 siRNA treatment on NFkB transcriptional activity as assessed by a GFP reporter construct (**C**). The effect of NFkB siRNA on NFkB mRNA levels (**D**) and the effect of NFkB siRNA in the presence of G3BP2 siRNA on NFkB transcriptional activity (**E**) and entry into the G_1_/S phase of the cell cycle (**F**). Data taken from Peluso et al. [87]. * indicates a value significantly different from scramble control.

PGRMC1 and PGRMC2 can localize the cytoplasm, mostly to the endoplasmic reticulum, where they interact with each other. They also form a complex that is composed of GTPase-activating protein-binding protein 2 (G3BP2). Depleting any of these three proteins from SIGCs results in entry into the cell cycle (Figure 5A,F) [87]. Interestingly, G3BP2 binds NFκB/p65 through an interaction with NFκB inhibitor alpha (IκBα), thereby maintaining NFκB/p65’s cytoplasmic localization and restricting its transcriptional activity. This conclusion is based on the findings that depleting either PGRMC1, PGRMC2, or G3BP2 increases NFκB transcriptional activity (Figure 5C) and leads to an increased entry into the cell cycle that is dependent on the presence of NFκB/p65 (Figure 5D–F) [87]. How progesterone affects this signal transduction pathway remains to be evaluated but it likely acts to maintain the integrity of the PGRMC:G3BP2:IκBα:NFκB complex within the cytoplasm.

PGRMC1 and PGRMC2 also interact with both small (40 s) and large (60 s) ribosomal proteins as well as eukaryotic translation initiation factors and eukaryotic elongation factors; all of which reside in the cytoplasm. More details on these interactions are presented in a recent publication [88]. Briefly, there are at least 10 ribosomal proteins that interact with both PGRMC1 and PGRMC2, while there are other ribosomal proteins that interact with either PGRMC1 or PGRMC2 [88]. These interactions suggest that PGRMCs are involved in organizing unique ribosomal complexes that could act to incorporate specific mRNAs and thereby determine in part which mRNAs are translated into proteins as proposed by Mauro and Edelman [89].

### 4.3. Nuclear Site of Action

The concept that the nuclear location of PGRMC1 is related to its mode of action is supported by the observation that in rapidly proliferating SIGCs PGRMC1 is located in both the cytoplasm and nucleus. As the rate of mitosis slows due to increased cell density, the amount of PGRMC1 localizing to the nucleus decreases with a corresponding increase in cytoplasmic PGRMC1. The change in cellular location corresponds to an increase in the proportion of monomeric form and a decrease in the proportion of oligomeric forms of PGRMC1 [77].

**Figure 6:**
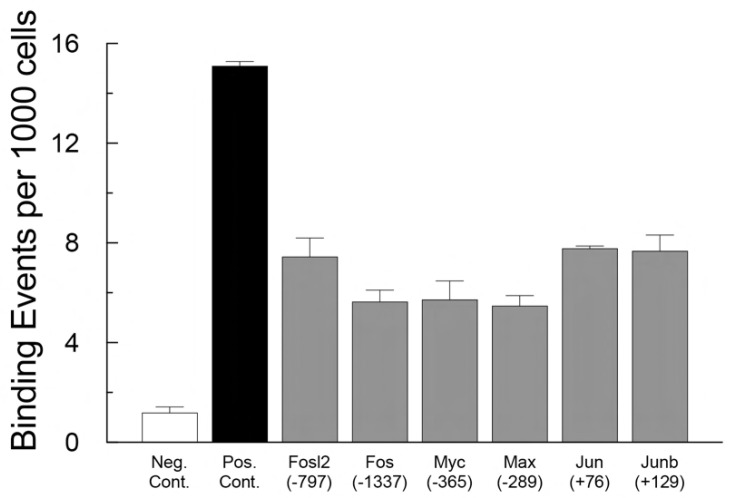
Specific binding of PGRMC1-Flag (i.e., >5 binding sites/1000 cells) to DNA segments located at or near the promoter region of several “immediate-early” genes associated with entry into the cell cycle. The gene and the distance from the start site that interacts with PGRMC1-Flag is shown on the X-axis. Analysis was completed using SIGCs and conducted by ActivMotif as outlined in their ChIP-seq (https://www.activemotif.com/catalog/819/chip-sequencing-service (accessed on 30 April 2022) and ChIP-qPCR services (https://www.activemotif.com/catalog/833/chip-qpcr-servicesaccessed on 30 April 2022).

It appears then that nuclear PGRMC1 is involved in regulating the rate of granulosa cell proliferation. The mechanism through which nuclear PGRMC1 mediates this action is unknown. There are at least two potential mechanisms by which nuclear PGRMC1 could influence entry into the cell cycle. The first mechanism could involve direct interaction with promoter regions of genes that stimulate cell cycle traverse such as c-myc and c-jun. To assess this possibility PGRMC1 was depleted from SIGCs using siRNA. The percentage of SIGCs incorporating BrdU after being treated with scramble siRNA was reduced by progesterone by 20 ± 0.5% but progesterone did not reduce the percentage of cells incorporating BrdU after being treated with PGRMC1 siRNA. To gain insight into how PGRMC1 influences the expression of genes that regulate entry into the cell cycle, cells were transfected for an expression vector encoding a Flag-tagged PGRMC1 and ChIP seq/ChIP-qPCR analysis was conducted. This analysis revealed that PGRMC1 interacted with the promoter regions of c-myc and c-jun as well as other members of the activating protein-1 (AP-1) family of transcription factors that promote entry into the cell cycle (Figure 6). Taken together, these data suggest that nuclear PGRMC1 interacts with the promoters of these AP-1 factors to suppress their expression and in turn entry into the cell cycle. That PGRMC1 predominately suppresses gene expression is also supported by microarray analysis that revealed that depletion of PGRMC1 in hGL5 cells disproportionately increased in the number of transcripts [76]. Moreover, a pathway analysis implicated PGRMC1 in the regulation of apoptosis, which is consistent with PGRMC1’s known biological action [76]. However, whether the PGRMC1 directly interacts with the promoter region of these immediate-early gene promoters or interacts with other nuclear proteins to function as an indirect transcriptional regulator that inhibits their expression is unknown at this point. Further PGRMC1 function within the nucleus may be dependent on the sumoylation and phosphorylation status of PGRMC1 since nuclear PGRMC1 is sumoylated [77] and phosphorylated [90].

## 5. PGRMCs and Follicle Growth In Vivo

**Figure 7:**
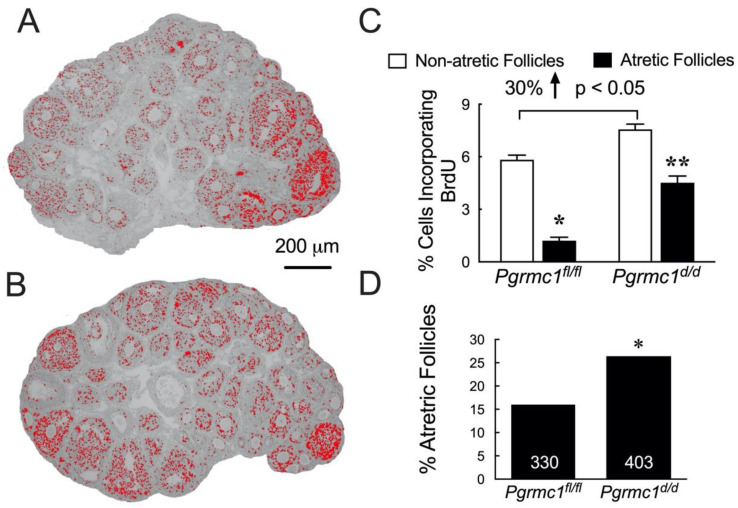
BrdU incorporation in the granulosa cells of nonatretic and atretic follicles of immature *Pgrmc1 ^fl/fl^* and *Pgrmc1 ^d/d^* mice. Panels (**A**) and (**B**) are low magnification images of the ovaries of *Pgrmc1 ^fl/fl^* and *Pgrmc1 ^d/d^* mice in which the cells incorporating BrdU are pseudo colored in red. Quantitative estimates of the percentage of granulosa cells incorporating BrdU in all sized non-atretic and atretic follicles of *Pgrmc1 ^fl/fl^* and *Pgrmc1 ^d/d^* mice is shown in panel (**C**) * indicates a significant decrease in atretic follicles compared to non-atretic follicles of *Pgrmc1 ^fl/fl^* mice; ** indicates a significant decrease in decrease in atretic follicles compared to non-atretic follicles of *Pgrmc1 ^d/d^* mice. The bracket refers to a 30% increase in percentage of cells incorporating BrdU in the *Pgrmc ^d/d^* mice compared to the *Pgrmc1 ^fl/fl^* mice. The percentage of nonatretic and atretic follicles/ovary is shown in panel (**D**) (* indicates a significant increase in the percentage of atretic follicles in *Pgrmc1 ^d/d^* mice compared to *Pgrmc1 ^fl/fl^* mice. Data taken from Peluso et al. [87].

The previously cited studies provide causal data demonstrating that progesterone acting through PGRMC1 and to some extent PGRMC2 regulates granulosa cell proliferation and viability in vitro. A remaining question relates to whether follicular development is dependent on PGRMC1 and PGRMC2. To address this, conditional knockout mice were developed in which the cre recombinase enzyme was expressed under the control of the anti-Mullerian hormone receptor type 2 promoter. In this immature mouse model, PGRMC1 was ablated in granulosa cells of developing follicles. This approach revealed that the number of antral follicles that developed in heterozygous knockout mice was not different from controls but the percentage of atretic follicles was significantly increased. In the homozygous PGRMC1 knockout mice the number of antral follicle was significantly reduced [91]. A more detailed study was also conducted in which BrdU was injected in PGRMC1 homozygous knockout mice to identify granulosa cells that were undergoing mitosis [87]. This study examined various sized follicles ranging from primary follicles through antral follicles. As can be seen by comparing overall BrdU incorporation within the ovaries of control *Pgrmc1 ^fl/fl^* mice (Figure 7A) to the ovaries of *Pgrmc1 ^d/d^* mice (Figure 7B), BrdU incorporation was increased in all sized follicles of the *Pgrmc1 ^d/d^* mice. Quantitative analysis revealed that 30% more granulosa cells entered the cell cycle as judged by BrdU incorporation in the *Pgrmc1 ^d/d^* mice (Figure 7C) and that this increase in BrdU incorporation was also observed in atretic follicles (Figure 7C). Importantly, the rate of follicular atresia was increased in the *Pgrmc1 ^d/d^* mice (Figure 7D). Since the progesterone levels were not affected by the ablation of PGRMC1 [38], these findings further support the concept that progesterone activation of PGRMC1 promotes the appropriate entry into the cell cycle such that granulosa cells complete mitosis, remain viable, and as a result promote the development of more and healthier antral follicles [92].

The previously cited studies did not assess changes in the number of primordial follicles. However, another study was conducted using a PGRMC1/PGRMC2 double knockout mouse in which cre recombinase enzyme was expressed under the control of the PGR promoter [93]. In this conditional knockout mouse, PGRMC1 and PGRMC2 were ablated in preovulatory follicles and the resulting corpora lutea. Follicle development was not altered in mice ≈3 months of age but at ≈6 months of age the number of primordial follicles present was reduced by about 80% of controls. This was likely due to changes in the corpora lutea that included an increased expression of vascular endothelial growth factor A (VEGFA). The ablation of PGRMC1 and PGRMC2 in the corpora lutea also resulted in changes in the levels of three factors that influence primordial follicle growth in the non-luteal component of the ovary: VEGFA, Kit ligand, and anti-Mullerian hormone. The expression levels of these three growth factors were different than that of the controls, which could account for the premature decrease in primordial follicle numbers. While this concepts requires additional testing, it is clear that the survival of primordial follicles is dependent in part on the ability of luteal cells to express PGRMC1 and PGRMC2 [93].

PGRMC1 may also play a role in the formation of primordial follicles. In this case, progesterone inhibits the formation of primordial follicles in cultured prenatal mouse ovaries with PGRMC1 being detected in both the primordial oocytes and associated pre-granulosa cells. Depleting PGRMC1 significantly reduced the mRNA levels and allowed for the formation of primordial follicles even in the presence of progesterone [94].

Collectively, these studies demonstrate that PGRMC1 can affect both the formation and survival of primordial follicles as well as affecting the rate at which the other sized follicle develop. How these two aspects of progesterone-PGRMC1 mediate these actions remains to be assessed but is likely dependent on the stage of ovarian development and the hormonal environment.

## 6. Summary and Future Research

Since its detection within the mammalian ovary in 2005, considerable progress has been made that demonstrates that PGRMC1 and its family member PGRMC2 play important and unique roles in progesterone’s ability to regulate of ovarian follicular growth. Although the idea that PGRMC1 binds progesterone was somewhat controversial, it is clear that PGRMC1 but not PGRMC2 not only binds progesterone but it mediates progesterone’s anti-mitotic and anti-apoptotic effects by activating several different signal transduction pathways. The ability to modulate these signal transduction pathways is dependent on both the ovarian cell type in which PGRMC1 and PGRMC2 are expressed as well as their cellular location which includes the plasma membrane, the membranes of cytoplasmic organelles, and the nucleus. Moreover, each cellular location likely involves specific interactions with numerous proteins that account for the diversity of progesterone-activated signal transduction pathways that are dependent on PGRMC1.

While these are some of the known components of progesterone-PGRMC signaling in the ovary, there remain many things that we do not know. These include but are not limited to:

1. What factors trigger the cell-cycle-dependent expression of PGRMC2 that appears to allow for the controlled entry into the cell cycle?
2. Do the interactions between PGRMC1, PGRMC2, PAQR7, and PGR result in a single complex or are there different combinations that occur at specific cellular sites and are these complexes linked to specific signal transduction pathways that are regulated by the hormonal environment?
3. How do the numerous potential post-translation modifications such as changes in sumoylation and phosphorylation affect PGRMC1′s cellular localization and interaction with specific proteins that ultimately accounts for progesterone’s actions?
4. Are there multiple mechanisms by which PGRMC modulates gene expression? If so, can these mechanisms be individually manipulated to either enhance or inhibit progesterone’s actions without influencing PGR’s role in ovarian function?
5. How are changes in the expression and sumoylation and phosphorylation status of PGRMC1 and PGRMC2 related to the dysfunction of the human ovary such as premature ovarian insufficiency and polycystic ovarian syndrome?
6. How does the expression profile of these progesterone receptors change or modulate progesterone’s ability to transduce its anti-mitotic and anti-apoptotic action and does the patient-specific profile affect the outcome of fertility treatment?

The answers to these questions will likely require more advanced imaging techniques and genetic tools. However, the answers to these questions could provide valuable insights into the complexities of progesterone’s action that could result in the development of specific antagonists and agonists that can modulate PGRMC’s actions without inferring with those progesterone-dependent actions mediated by PGR and/or PAQRs. If so, these putative progesterone modulators could find application not only as related to enhancing or inhibiting fertility but also for the treatment of ovarian and other cancers that express elevated levels of PGRMC1 [90].

## Data Availability

Not applicable.

## Abbreviations

PGR	Nuclear Progesterone Receptor
PAQR	Progestin and AdipoQ Receptor
PGRMC	Progesterone Membrane Receptor Component family
SIGCs	Spontaneously Immortalized Granulosa Cells
PAIRBP1	Plasminogen Activator Inhibitor 1 RNA-binding protein
SERBP1	Serpine mRNA Binding Protein 1
IVF	In Vitro Fertilization
PCOS	Polycystic Ovary Syndrome
H165R	A missense point mutation where histidine (H) at amino acid 165 is changed to an arginine (R)
TCF/Lef1	Ternary Complex Factors/Lymphoid enhancer factor 1
G3BP2	GTPase-activating protein-binding protein 2
NFκB/p65	Nuclear Factor Kappa B
IκBα	NFκB inhibitor alpha
VEGFA	Vascular Endothelial Growth Factor A
EGF	Epidermal growth factor
BrdU	Bromodeoxyuridine/5-bromo-2’-deoxyuridine
AP-1	Activating protein 1
PI3K	Phosphoinositide 3-kinase
PKB	Protein kinase B (PKB; as known as AKT)
MAPK	Mitogen-activated protein kinase
